# New Books

**Published:** 2004-12

**Academia to Biotechnology: Career Changes at any Stage**

Jeffrey Gimble

Burlington, MA:Elsevier, 2004. 186 pp. ISBN: 0-12-284151-4, $34.95

**Cancers in the Urban Environment**

Thomas Mack

Burlington, MA:Academic Press, 2004. 656 pp. ISBN: 0-12-464351-5, $99.95

**Cell Cycle Control: Mechanisms and Protocols**

Tim Humphrey, Gavin Brooks

Totowa, NJ:Humana Press, 2004. 416 pp. ISBN: 1-58829-144-8, $99.50

**Climate Change, Policy, and Global Trade**

Christoph Böhringer, Andreas Löschel, eds.

New York:Springer-Verlag, 2004. 381 pp. ISBN: 3-7908-0171-2, $79.95

**Drug Metabolism and Transport: Molecular Methods and Mechanisms**

Lawrence H. Lash

Totowa, NJ:Humana Press, 2004. 430 pp. ISBN: 1-58829-324-6, $125

**Handbook of Bioethics: Taking Stock of the Field from a Philosophical Perspective**

George Khushf, ed.

New York:Springer-Verlag, 2004. 574 pp. ISBN: 1-4020-1870-3, $257

**Handbook of Immunohistochemistry and in Situ Hybridization of Human Carcinomas**

M. Hayat, ed.

Burlington, MA:Elsevier, 2004. 400 pp. ISBN: 0-12-333941-3, $169.95

**Immunochemical Protocols, 3rd ed.**

Robert Burns

Totowa, NJ:Humana Press, 2004. 325 pp. ISBN: 1-58829-274-6, $99.50

**Immunology Guidebook**

Julius Cruse, Robert Lewis, Huan Wang, eds.

Burlington, MA:Academic Press, 2004. 1,000 pp. ISBN: 0-12-198382-X, $125

**Man-Made and Natural Radioactivity in Environmental Pollution and Radiochronology**

Richard Tykva, Dieter Berg, eds.

New York:Springer-Verlag, 2004. 428 pp. ISBN: 1-4020-1860-6, $149

**Mechanisms of Carcinogenesis: Contributions of Molecular Epidemiology**

P. Bufffler, J. Rice, M. Bird, P. Boffetta, eds.

Lyon, France:IARC Press, 2004. 460 pp. ISBN: 92-832-2157-5, $40

**Metals in Society and in the Environment: A Critical Review of Current Knowledge on Fluxes, Speciation, Bioavailability and Risk for Adverse Effects of Copper, Chromium, Nickel and Zinc**

Lars Landner, Rudolf Reuther

New York:Springer-Verlag, 2004. 407 pp. ISBN: 1-4020-2740-0, $99

**Methods in Modern Biophysics**

Bengt Nölting

New York:Springer-Verlag, 2004. 254 pp. ISBN: 3-540-01297-4, $59.95

**Pathology and Genetics of Tumours of the Lung, Pleura, Thymus and Heart**

W.D. Travis, E. Brambilla, H.K. Müller-Hermelink, C.C. Harris, eds.

Lyon, France:IARC Press, 2004. 344 pp. ISBN: 92-832-2418-3, $110

**Pursuing the Endless Frontier: Essays on MIT and the Role of Research Universities**

Charles M. Vest

Cambridge, MA:MIT Press, 2004. 200 pp. ISBN: 0-262-22072-5, $24.95

**Regulatory Mechanisms of Intracellular Membrane Transport**

Sirkka Keränen, Jussi Jäntti, eds.

New York:Springer-Verlag, 2004. 214 pp. ISBN: 3-540-22302-9, $149

**Risk Assessment as a Tool for Water Resources Decision-Making in Central Asia**

Christopher M. Teaf, Bulat K. Yessekin, Mikhail Kh. Khankhasayev, eds.

New York:Springer-Verlag, 2004. 338 pp. ISBN: 1-4020-1840-1, $154

**Scientific Papers and Presentations, 2nd ed.**

Martha Davis

Burlington, MA:Elsevier, 2004. 384 pp. ISBN: 0-12-088424-0, $29.95

**Stem Cells in Development and Disease**

Gerald Schatten

Burlington, MA:Academic Press, 2004. 270 pp. ISBN: 0-12-153160-0, $149.95

**The Laboratory Mouse**

Hans Hedrich, ed.

Burlington, MA:Academic Press, 2004. 656 pp. ISBN: 0-12-336425-6, $199.95

**Transcription Factors**

Manfred Gossen, Jörg Kaufmann, Steven J. Triezenberg, eds.

New York:Springer-Verlag, 2004. 581 pp. ISBN: 3-540-21095-4, $399

